# Cerium‐Organic Framework UiO‐66(Ce) as a Support for Nanoparticulate Gold for Use in Oxidation Catalysis

**DOI:** 10.1002/asia.202401035

**Published:** 2024-10-31

**Authors:** Baiwen Zhao, Reza J. Kashtiban, Steven Huband, Marc Walker, Richard I. Walton

**Affiliations:** ^1^ Department of Chemistry University of Warwick Gibbet Hill Road Coventry CV4 7AL UK; ^2^ Department of Physics University of Warwick Gibbet Hill Road Coventry CV4 7AL UK

**Keywords:** MOF, Heterogeneous catalyst, UiO-66, Au nanoparticles, Alcohol oxidation

## Abstract

An optimised synthesis of the metal‐organic framework (MOF) UiO‐66(Ce) is reported using a modulator‐free route, yielding ~5 g of material with high crystallinity and 22 % ligand defect. Two methods are developed for loading gold nanoparticles onto the MOF. The first uses a double‐solvent method to introduce HAuCl_4_ onto UiO‐66(Ce), followed by reduction under 5 % H_2_ in N_2_, while the second is a novel one‐pot method where HAuCl_4_ is added to the synthesis mixture, forming Au nanoparticles within the pores of the UiO‐66(Ce) during crystallisation. Analysis using powder X‐ray diffraction (PXRD), nitrogen adsorption isotherms, transmission electron microscopy and small‐angle X‐ray scattering (SAXS) reveals that the two‐step double‐solvent method yields gold crystallites on the external surface of the MOF particles that are visible by PXRD. In contrast, the one‐pot method forms smaller gold crystallites, with a distribution of sizes centred on ~4 nm diameter as seen by SAXS, with evidence from PXRD for the smallest particles being present within the MOF structure. The Au‐loaded UiO‐66(Ce) materials are evaluated for the catalytic oxidation of vanillyl alcohol to vanillin at 60 °C. Our findings indicate that incorporating Au nanoparticles via the one‐pot synthesis method, enhances redox activity, achieving 43 % conversion and 90 % selectivity towards vanillin.

## Graphical abstract

A one‐pot route via addition of HAuCl_4_ to the solvothermal crystallisation of UiO‐66(Ce) MOF gives well‐dispersed gold nanoparticles, with spectroscopic evidence of a Au−Ce interaction, leading to catalytic activity for vanillyl alcohol oxidation.

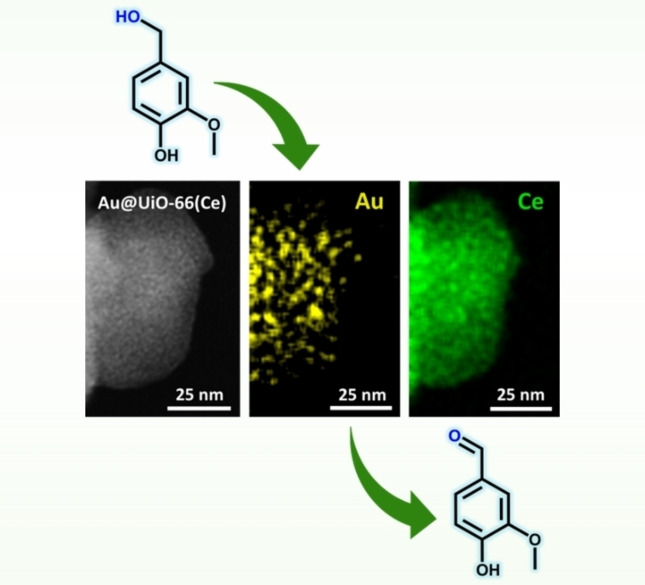

## Introduction

The combination of metal nanoparticles (NPs) and high‐surface‐area materials such as porous carbon, zeolites and MOFs attracts significant interest and offers unique opportunities for enhancing catalytic performance in terms of activity, selectivity and stability.[^1^, ^2^] Porous materials offer advantages over traditional oxide materials because of their enhanced surface area and tailored pore structures that provide access to greater number of active sites for catalytic reactions, and also the possibility of shape‐ and size‐selectivity. This enables precise control over catalytic activity and selectivity. The incorporation of noble‐metal NPs within MOFs has been a subject of intense recent research since it provides an efficient approach to stabilise and utilise NPs for various applications.^[3]^ The tunable pore size of MOFs is particularly advantageous for noble‐metal@MOF composites. By controlling the synthesis parameters, such as the choice of metal nodes, organic linkers, and reaction conditions, the pore size and shape of MOFs can be tailored.[^4^, ^5^]

Au@CeO_2_ is an important catalyst for redox catalysis.^[6]^ This catalyst shows high activity for the water gas shift (WGS) reaction^[7]^ and CO oxidation.^[8]^ It is also a highly active and selective heterogeneous catalyst for the aerobic oxidation of primary alcohols to aldehydes.[^9^, ^10^] Importantly, the reversible shifting between Ce^4+^ and Ce^3+^ oxidation states enables the materials to store or donate oxygen during redox reactions.^[11]^ Gold nanoparticles strongly interact with the oxide support, creating oxygen vacancies that provide active sites for the adsorption and activation of reactant molecules, enhancing the co‐reactivity of both CeO₂ and gold in oxidation reactions.^[12]^ As Au NPs interacting with oxide supports result in higher catalytic activity, the metal‐organic framework UiO‐66 could also be an advantageous support because the building units consist of Ce/Zr−O clusters.^[13]^ UiO‐66 is a well‐known MOF constructed from *M*_6_O_4_(OH)_4_ building units (*M*=tetravalent metal) connected in three dimensions by benzene‐1,4‐dicarboxylate ligands to yield a porous network that possesses unusual structure stability.^[14]^ The Ce/Zr−O clusters within the UiO‐66 framework can potentially provide oxygen vacancies and Lewis acid sites, to promote catalytic reactions and interact with the Au NPs.

A Au@UiO‐66(Zr) catalyst for CO oxidation was developed where zirconium UiO‐66 support was synthesised first, followed by loading gold nanoparticles using oleylamine as a reducing and stabilising agent.^[15]^ Another Au@UiO‐66(Zr) material was studied as a catalyst in the oxidation of benzyl alcohol and benzyl amine.^[16]^ Different methods using NaBH_4_, triethylamine and H_2_ have been studied to reduce the gold precursors added to the MOF host. A core‐shell structure Pt/Au@Pd@UiO‐66(Zr) catalyst was reported for CO_2_ hydrogenation to produce CO.^[17]^ The material was prepared by one‐step hydrothermal synthesis using polyvinylpyrrolidone (PVP) to control the size and stabilise the Au NPs. Au@UiO‐66(Zr) has also been developed for metal detection,^[18]^ electrochemical sensing,^[19]^ and photocatalysis.^[20]^ Considering UiO‐66(Ce), to our knowledge there is only one report on its use as a support for Au clusters, where it was used as a catalyst for the oxidation of cinnamyl alcohol.^[21]^ However, it is worth noting that a direct one‐step synthesis of a cerium‐based Au@UiO‐66(Ce) material, which would streamline the immobilisation of Au NPs directly to the MOF structure during the initial synthesis process, has not yet been reported.

Vanillin (4‐hydroxy‐3‐methoxybenzaldehyde) is a highly significant chemical that finds extensive use in various industries, including food additives, pharmaceuticals, and cosmetics.^[22]^ It also serves as an versatile precursor for fine chemicals due to its two reactive groups, namely phenolic‐OH and aldehyde, which can be further functionalised.^[23]^ This characteristic makes vanillin a promising candidate as a renewable aromatic building block. It is worth noting that the production of vanillin from lignin biomass exceeds 3000 tons annually, highlighting its importance in the industry.^[24]^ One of the methods to produce vanillin is oxidation of lignin and lignin‐derived alcohols using heterogeneous catalysis.^[25]^ Vanillyl alcohol (4‐hydroxy‐3‐methoxybenzyl alcohol) is an important phenolic compound derived from lignin and its selective oxidation to produce vanillin has been widely studied using a variety of heterogeneous catalysts.^[26]^

Here, we report the synthesis of Au@UiO‐66(Ce) via a one‐pot method, comparing it with a double‐solvent impregnation method from a pre‐made sample of UiO‐66(Ce) where the reduction of Au^3+^ ions to Au nanoparticles under H_2_ in N_2_ on a UiO‐66(Ce) support is performed. The catalytic oxidation activity was evaluated through the oxidation of vanillyl alcohol to vanillin.

## Results and Discussion

### Synthesis of UiO‐66(Ce)

**Figure 1:**
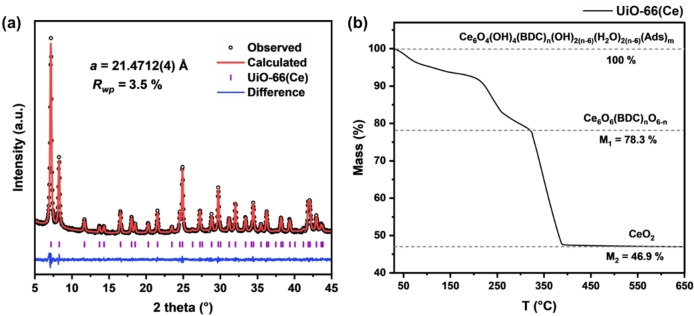
(a) PXRD pattern of the scaled‐up synthesised UiO‐66(Ce) analysed using a Pawley fit (space group *Fm* $\bar{3}$ *m*), (b) TGA trace in air of scaled‐up synthesised UiO‐66(Ce), Ads=adsorbed H_2_O, DMF etc.

To serve as a suitable support for Au NP loading, UiO‐66(Ce) was prepared prior to the nanoparticle loading process. For consistency in the experiment, the synthesis, based on a reported route,^[27]^ was scaled up to yield up to 5 g of material using a 250 mL solvothermal reactor. The PXRD patterns (Figure 1(a)) were fitted using a unit cell in the *Fm* $\bar{3}$ *m* space group, consistent with the expected structure without showing extra peaks indicative of ordered defects. The fitted lattice parameter for the scaled‐up synthesised UiO‐66(Ce) is 21.4712(4) Å, close to the reported value of 21.4727 Å.^[27]^ The ligand defect of the sample was determined by thermogravimetric analysis (TGA), as shown in Figure 1(b), with the chemical composition and mass percentage at each step. By comparing the mass percentages before and after the final ligand decomposition phase, the ligand defect content was calculated to be 22.1 %, indicating that 1.3 in 6 BDC ligands were missing. The level of ligand defects is in the range reported by other for UiO‐66(Ce) materials. For example, Lammert *et al*. found 0.5 out of 6 ligands on average were missing,^[27]^ Islamoglu *et al*. found 1.6 missing per node,^[28]^ while González *et al*. found 1.9 out of 6 missing.^[29]^ The number of ligand defects will depend on the precise method of synthesis, for example whether a modulator was used. The synthesised UiO‐66(Ce) shows microporosity, characterised by Type‐I isotherms,^[30]^ as shown in Figure S5. The BET surface area and pore volume for the sample are 827 m^2^/g and 0.32 cm^3^/g, respectively.

### Double‐Solvent Method to Immobilise Au NPs

The double‐solvent method, was based on a method reported for incorporating oxides into mesoporous silica,^[31]^ and has been used previously for the immobilisation of Pt and Au NPs within the pores of MOFs, preventing aggregation on external surfaces.[^32^, ^33^] This method leverages the immiscibility of hydrophilic and hydrophobic solvents. In UiO‐66, defects in the form of missing linkers render this MOF hydrophilic,[^34^, ^35^] and therefore, to immobilise Au NPs onto UiO‐66(Ce), a hydrophilic solution (water) containing the precious metal precursor is introduced into a hydrophobic suspension (hexane) of the adsorbent under vigorous stirring. This results in the formation of dispersed droplets containing the precursor molecules, which are uniformly introduced into the hydrophilic sites of UiO‐66(Ce). The aim is that the aqueous solution containing the Au precursor is predominantly confined within the pores of the MOF material, leading to well dispersed precursor for nanoparticles.^[36]^

**Figure 2:**
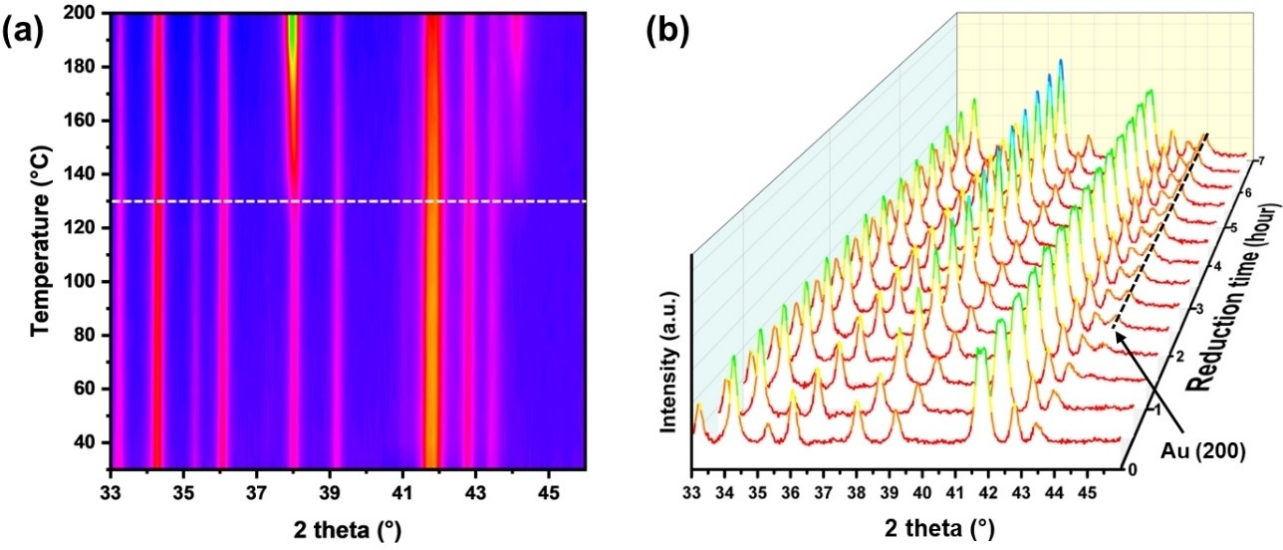
(a) Contour plot for VT‐PXRD patterns of the 3 %HAuCl_4_/UiO‐66(Ce) sample heated from 30 °C to 200 °C under a gas flow of 5 % H_2_ in N_2_. The dashed line indicates the start of the reduction of Au^3+^ to form Au NPs. (b) Plot of VT‐PXRD patterns of 3 %HAuCl_4_/UiO‐66(Ce) under 5 % H_2_ in N_2_ following reduction at a constant temperature of 130 °C for up to 7 hours.

The reduction of HAuCl_4_ precursor was conducted by heating the precursor HAuCl_4_/UiO‐66(Ce) in a 5 % H_2_ in N_2_ gas mixture. The optimal condition was established by using variable‐temperature PXRD (VT‐PXRD). Figure 2(a) shows the 3 %HAuCl_4_/UiO‐66(Ce) sample under reduction atmosphere heated from 30 to 200 °C in 5 % H_2_ in N_2_. The formation of crystalline Au nanoparticles is indicated from diffraction peaks at 2θ=38.2° (111) and 44.4° (200), and show that the reduction of Au^3+^ begins at approximately 130 °C. To determine the optimal duration for the reduction process, VT‐PXRD patterns of the 3 %HAuCl_4_/UiO‐66(Ce) were also collected at constant temperature of 130 °C under 5 % H_2_ in N_2_, Figure 2(b). The (200) peak for the crystalline Au appears at 2θ=44.4° after 2 hours. After 7 hours, the Au nanoparticle peaks become more intense and sharper, indicating aggregation and larger Au crystallite formation. Based on the observed changes in the PXRD patterns, the optimal condition to reduce the Au‐loaded UiO‐66(Ce) was determined to be 130 °C for 2 hours. This ensures a mild reduction, forming Au NPs with small size. X‐ray fluorescence (XRF) analysis provides a Au loading of 3.20 wt %, which is consistent to the intended value.

**Figure 3:**
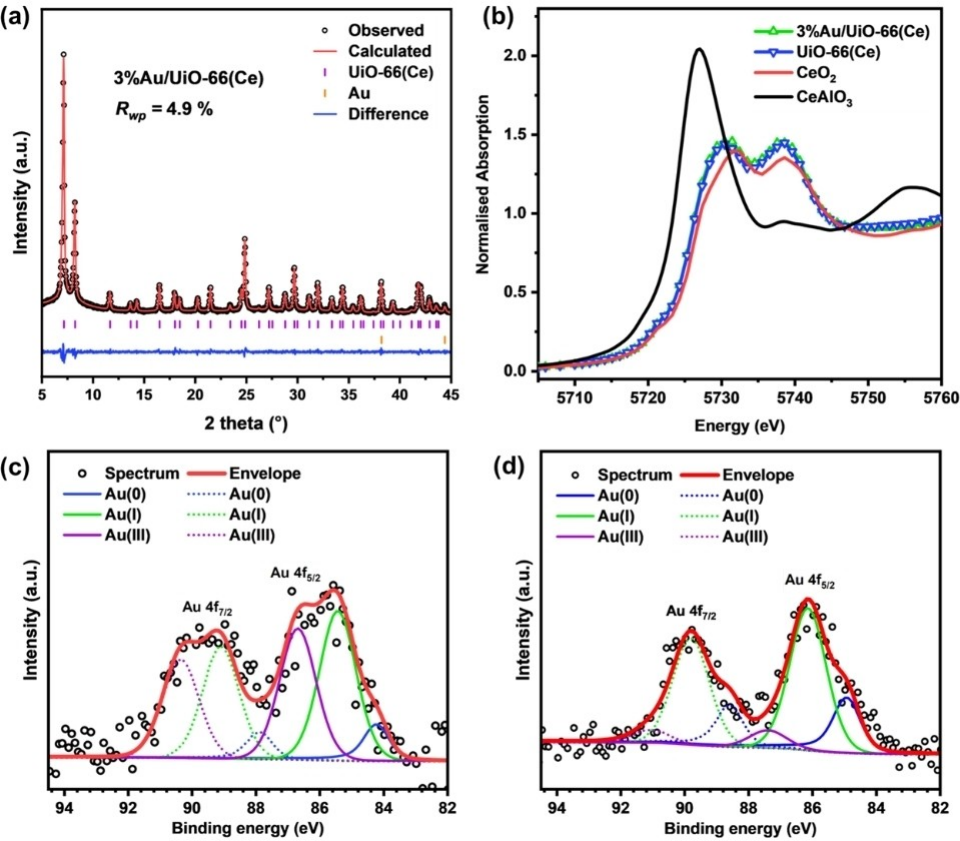
(a) PXRD pattern 3 %Au/UiO‐66(Ce) analysed using a Pawley fit with two phases (space group *Fm* $\bar{3}$ *m* indexed for UiO‐66(Ce) and Au)). (b) Normalised Ce L_III_‐edge XANES spectra of the blank MOF and 3 %Au/UiO‐66(Ce). The Ce(IV) and Ce(III) reference materials are CeO₂ and CeAlO₃, respectively. (c) XPS spectra of Au 4 f for 3 %HAuCl_4_/UiO‐66(Ce). (d) XPS spectra of Au 4 f for 3 %Au/UiO‐66(Ce).

Figure 3(a) shows the PXRD pattern 3 %Au/UiO‐66(Ce) refined using a Pawley fit. The similarity in crystallinity confirms the stability of the MOF after the impregnation and reduction process. The presence of a small peak corresponding to the (200) Bragg peak of Au can be observed in the PXRD pattern of the 3 %Au/UiO‐66(Ce). This peak indicates the successful reduction of Au^3+^ ions to Au NPs. The Au is indexed to the space group *Fm* $\bar{3}$ *m* with a lattice parameter of 4.0804(4) Å. The UiO‐66(Ce) is also fitted to a cubic unit cell with *a*=21.4926(4) Å, which is slightly larger than the fitted lattice parameter for the unmodified UiO‐66(Ce) of 21.4712(4) Å. While the difference between these lattice parameters is small, it may be due to differing levels of ligand defects between the materials, as well as the effect of loading gold. The PXRD patterns (Figure S4) of 0.5 %Au/UiO‐66(Ce) show no gold diffraction peaks, so the pattern was fitted to a single UiO‐66 phase, likely due to the low Au loading. TG curves (Figure S1–S2) reveal that the samples prepared by double‐solvent/reduction method exhibit similar decomposition temperatures and mass loss for the organic ligand compared to the UiO‐66(Ce) MOF, indicating that the sample was not subject to structural defects during the reduction process.

Ce L_III_‐edge X‐ray absorption near edge structure (XANES) characterised the oxidation state of Ce in the MOFs before and after the reduction process, as shown in Figure 3(b). CeAlO_3_ and CeO_2_ served as references for Ce(III) and Ce(IV), respectively. These results show that the reduction converts Au^3+^ to metallic Au NPs without altering the Ce oxidation state. The spectra indicate that Ce is predominantly in the Ce(IV) state with only a minor amount of Ce(III) suggested by a left shift in the near‐edge feature from CeO_2_ in both the blank MOF and 3 %Au/UiO‐66(Ce). Previous studies have reported that mixed‐valence Ce(IV)/Ce(III) cations can be present in the secondary clusters of Ce‐based UiO‐66 materials.[^37^, ^38^] XPS analysis was conducted to examine the oxidation state of Au on the surface of UiO‐66(Ce) before (Figure 3(c)) and after (Figure 3(d)) reduction. Binding energies of Au 4f_7/2_ at 84.9, 86.3, and 87.4 eV correspond to Au⁰, Au^+^, and Au^3+^, respectively. The proportion of Au^3+^ relative to total amount of Au (Table S3) decreased from 41 % to 8 % after being treatment under 5 % H_2_ in N_2_ consistent the with intended reduction of the precursor. The fact that some Au^3+^ remains, along with Au^+^ signifies an interaction between metallic gold and the [Ce_6_O_4_(OH)_4_]^12+^ cluster that involves a charge transfer from Au^0^ to Ce^4+^, leading to the formation of Au^+^ and Ce^3+^.[^39^, ^40^] The presence of cationic gold species (Au^+^ and Au^3+^) aligns well with previously reported observations on Au‐CeO_2_ materials,[^41^, ^42^, ^43^] where XPS has been similarly been used to detect such an interaction and indicates that UiO‐66(Ce) as a support exhibits similar electronic interactions. The Ce 3d XPS spectra (Figure S9) suggest the presence of Ce^3+^ at the surface, which accounts also for the small amount of Ce^3+^ detected in the XANES analysis

**Figure 4:**
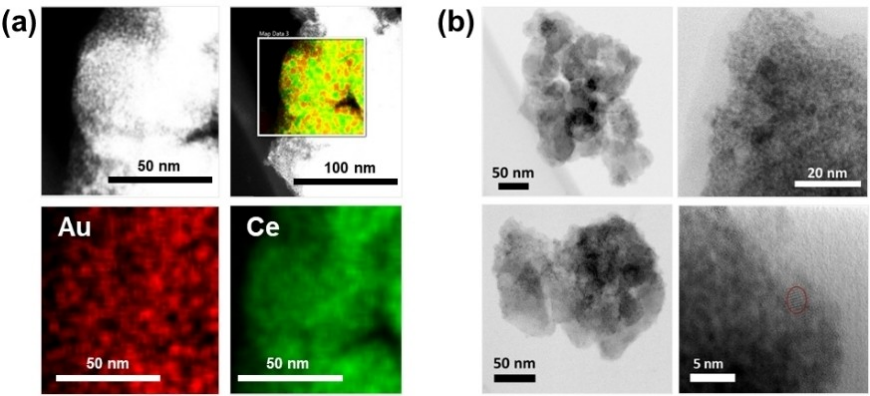
(a) ADF‐STEM image and overlayed STEM‐EDX composite map with individual Au and Ce element maps, (b) HR‐TEM images of 3 %Au/UiO‐66(Ce), where the circled region suggests the presence of small Au nanoparticles of 3 nm.

The N₂ sorption isotherms at 77 K (Figure S6) reveal a decreased saturated N₂ uptake for 3 %Au/UiO‐66(Ce) compared to the UiO‐66(Ce) MOF. Both samples exhibit microporosity, characterised by Type‐I isotherms. The BET surface area and pore volume (Table S2) for 3 %Au/UiO‐66(Ce) are 368 m^2^/g and 0.15 cm^3^/g, respectively, while the blank UiO‐66(Ce) shows a BET surface area of 827 m^2^/g and a pore volume of 0.32 cm^3^/g. The significant reduction in porosity indicates that the micropores are occupied by the Au NPs blocking some channels and reducing the surface area accessible for nitrogen adsorption. Scanning transmission electron microscopy – energy dispersive X‐ray spectroscopy (STEM‐EDX) mapping (Figure 4(a)) and high‐resolution transmission electron microscopy (HR‐TEM) images (Figure 4(b)) were obtained to provide detailed information about the distribution of Au nanoparticles within the MOF and their nanoscale size. The EDX mapping analysis indicates that both Ce and Au are evenly distributed throughout the material, suggesting no phase separation or clustering of either element. HR‐TEM images reveal small Au nanoparticles dispersed on the MOF surface, approximately 3 nm in diameter, along with larger particles more than 20 nm in size. The sample exhibits a particle size range of 3–25 nm, and the larger particles may result from agglomeration due to the heating used in the reduction step. The detection of Au in the PXRD pattern of the higher‐loaded sample is consistent with the presence of crystallites of NPs above 10 nm in diameter, as observed in Au/UiO‐66(Zr) samples.[^20^, ^44^]

### One‐Pot Synthesis of Au@UiO‐66(Ce)

**Figure 5:**
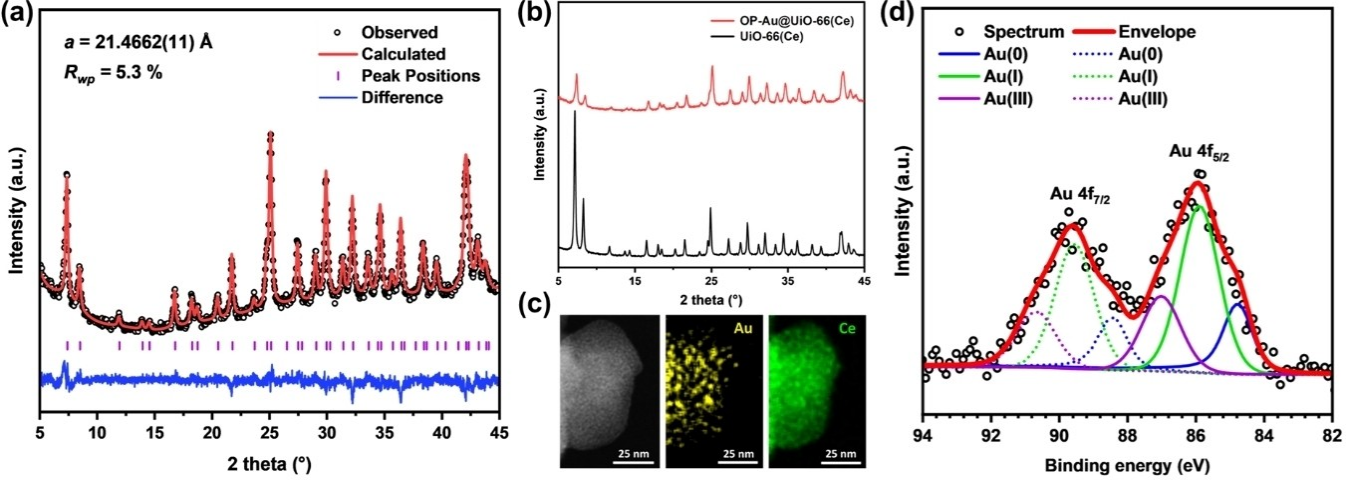
(a) PXRD pattern of one‐pot synthesised OP−Au@UiO‐66(Ce) analysed using a Pawley fit (space group *Fm* $\bar{3}$ *m*), (b) Comparison of the PXRD patterns of OP−Au@UiO‐66(Ce) and UiO‐66(Ce), (c) ADF‐STEM image (left), STEM‐EDX elemental maps with Au (middle) and Ce (right) dispersion. (d) XPS spectra of Au 4 f for OP−Au@UiO‐66(Ce).

The exact gold loading of the one‐pot synthesised sample, OP−Au@UiO‐66(Ce), was determined by XRF analysis (Table S1) showing the Au loading to be 3.86 wt %. The formation of the OP−Au@UiO‐66(Ce) was confirmed through PXRD analysis, as displayed in Figure 5(a). The PXRD patterns were fitted using a unit cell in the *Fm* $\bar{3}$ *m* space group, consistent with the expected structure for UiO‐66(Ce).^[45]^ The results are in good agreement with the reported UiO‐66 structure, showing no evidence of extra peaks that might indicate the presence of ordered defects. The fitted lattice parameter for the synthesised UiO‐66(Ce) is 21.4662(11) Å, which is close to the reported value of 21.4727 Å.^[27]^ The PXRD pattern (Figure 4(b)) shows a significant decrease in the intensity of peaks observed at low 2θ angles, ranging from 5° to 10° compared to the as‐made UiO‐66(Ce) material. This reduction in peak intensity can be attributed to the incorporation of Au nanoparticles within the pore structures of the MOF. This phenomenon, observed in previous studies involving the incorporation of guest species into porous materials, confirms that filling the pores with nanoparticles or other substances significantly attenuates diffraction peaks corresponding to large interplanar spacings.^[46]^ It is noteworthy that for this material there are no Bragg peaks of crystalline gold, in contrast to the sample discussed above, which suggests that the gold crystallites prepared by this one‐pot method are considerably smaller. Elemental analysis by TEM‐EDX mapping (Figure 4(c)), indicates the distribution of Au and Ce within the MOFs. The elemental mapping demonstrates that both Au and Ce are uniformly dispersed throughout the materials, with no significant separation or agglomeration observed. The Au 4 f XPS analysis (Figure 5(d)) reveals mixed oxidation states of Au on the surface of the OP−Au@UiO‐66(Ce) sample, similar to that observed in the 3 %Au/UiO‐66(Ce) sample. Binding energies of Au 4f_7/2_ at 84.8, 85.9, and 87.0 eV are indicative of Au⁰, Au^+^, and Au^3+^, respectively, with the proportions of 26 %, 51 % and 23 %. This mixed oxidation state is attributed to the interaction between Au⁰ and Ce⁴^+^,[^39^, ^40^, ^42^] leading to the oxidised of Au^+^ and Au^3+^ and the concurrent reduction of Ce⁴^+^ to Ce^3+^, as evidenced by the Ce 3d XPS spectra (Figure S10).

The TGA curves of the sample, collected under heating in air up to 700 °C, are presented in Figure S3. The OP−Au@UiO‐66(Ce) sample exhibits similar thermal stability to UiO‐66(Ce), with ligand decomposition occurring at approximately 320 °C. This indicates that the incorporation of Au nanoparticles into the MOF does not significantly affect the overall thermal stability of the MOF structure. Prior to the measurements, the samples were degassed at 120 °C under vacuum for 4 hours to remove any adsorbed gases or volatile impurities. The isotherms at 77 K obtained from N₂ sorption measurements, shown in Figure S7, reveal the presence of microporosity in the samples. The BET surface areas of OP−Au@UiO‐66(Ce) and UiO‐66(Ce) are 680 m^2^/g and 827 m^2^/g, respectively, with corresponding micropore volumes of 0.26 cm^3^/g and 0.33 cm^3^/g. The BET surface area of OP−Au@UiO‐66(Ce) is approximately 18 % lower than that of the blank MOF, and the adsorbed quantity from the isotherms is significantly reduced. The decreased sorption capacity in OP−Au@UiO‐66(Ce) is consistent with Au nanoparticles occupying the micropores, which reduces the available surface area and pore volume.

**Figure 6:**
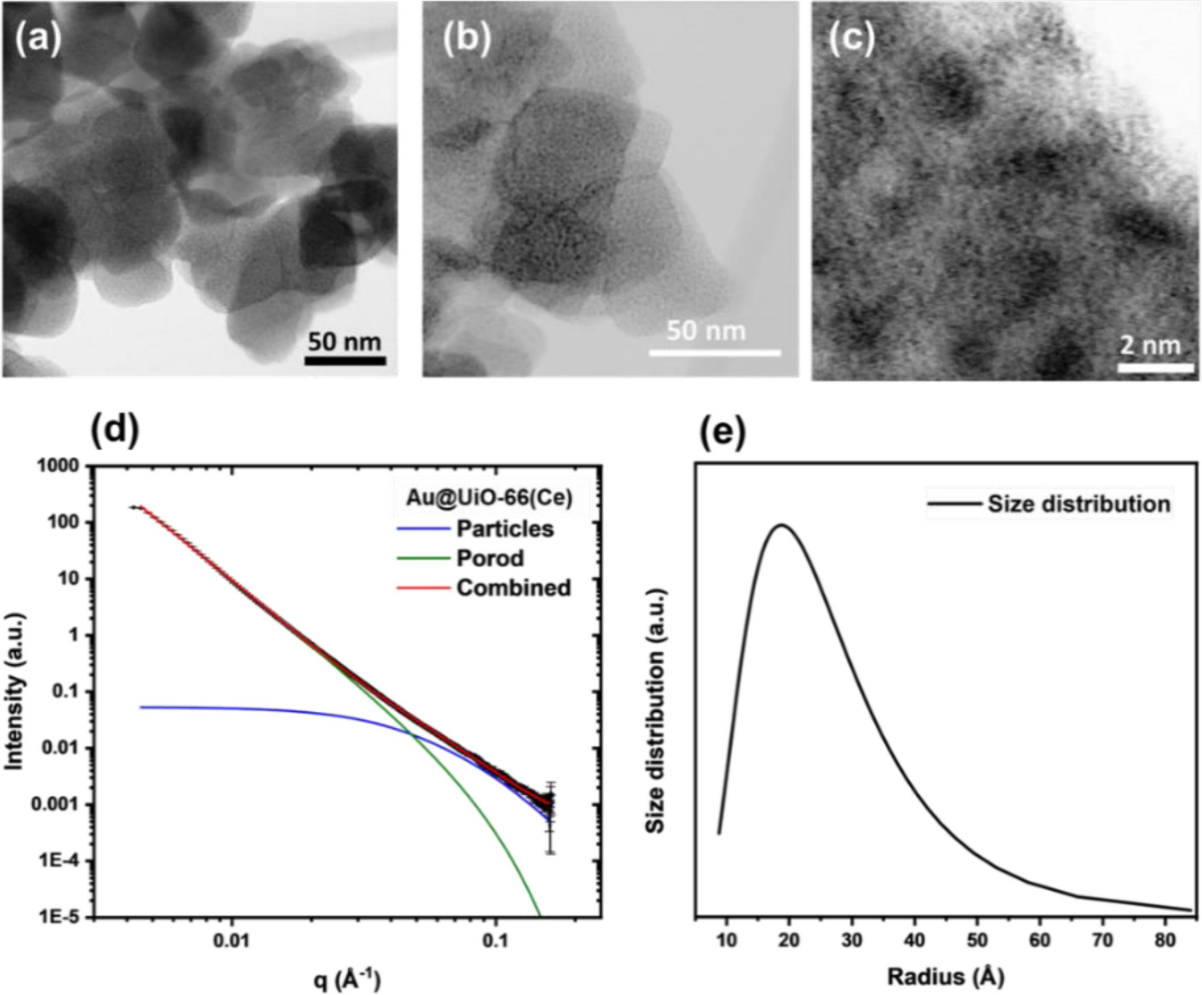
(a)–(c) High‐resolution TEM images of OP−Au@UiO‐66(Ce), (d) SAXS scattering signal plot and the fitting of the data, with the model for particles larger than 140 nm (green line), nanomaterials (blue line) and the overall scattering (red line), (e) Particle size distribution in radius derived from a spherical model for OP−Au@UiO‐66(Ce), showing mode particle radius of 2.2 nm.

Figure 6(a–c) present HR‐TEM images of the OP−Au@UiO‐66(Ce) sample. It can be seen that that the particles exhibit a cubic morphology, with an average crystallite size of approximately 50 nm. The images reveal small Au NPs in the sample, approximately 2 nm in diameter, and no large agglomeration of Au species was observed. SAXS measurements were used to provide information about the average particle size of gold present in the sample, complementing the data obtained from TEM imaging. The SAXS data indicates that the scattering signal is predominantly contributed by MOF particles larger than 140 nm. This observed size is inflated due to particle compression in the powder sample and does not represent the actual particle size of the MOF. However, a minor scattering signal from NPs in the sample was observed. Figure 6(d) illustrates the measured scattering signal. To model the scattering originating from particles larger than 140 nm, a Porod gradient was employed, allowing for the separation of the scattering contributions from larger particles and smaller nanoparticles, as indicated by the green line in the figure. At a q value of approximately 0.05 Å^−1^, a discernible deviation from the linear Porod line is evident, indicating the presence of scattering from nanoparticles. To accurately account for this scattering, a spherical model was used. The scattering contribution from the nanoparticles is represented by the blue line in the figure. The overall scattering, incorporating both the nanoparticles and the MOF particles larger than 140 nm, is displayed by the red line. The analysis of the scattering curve allows for the determination of the mean particle radius. As shown in Figure 6(e), the fitting demonstrates a mode particle radius of 2.2 nm. It may be noted that SAXS analysis of the samples prepared by the alternative double‐solvent method could not detect any well‐defined small scattering objects, consistent with the agglomeration of Au in those materials.

### Vanillyl Alcohol Oxidation

**Scheme 1:**
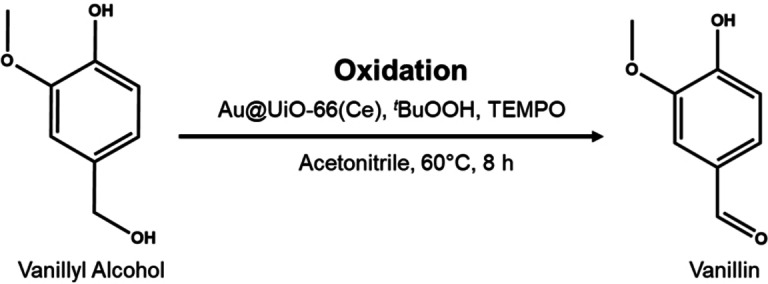
Catalytic conversion of vanillyl alcohol to vanillin.

The reaction scheme of selective vanillyl alcohol oxidation is shown in Scheme 1. Based on previous studies,[^27^, ^47^] acetonitrile was chosen as the solvent. The inclusion of 2,2,6,6‐tetramethylpiperidine‐1‐oxyl (TEMPO) radical as a cocatalyst was used to promote the oxidation of primary alcohols to aldehydes.[^48^, ^49^] Previous studies have reported the effective use of TEMPO in the oxidation of vanillyl alcohol to vanillin.[^26^, ^50^] The oxidant used was *tert‐*butyl hydroperoxide (^*t*^BuOOH) which was chosen as it is considered to be relatively environmentally friendly.^[51]^ Upon degradation, ^*t*^BuOOH primarily produces water and *tert*‐butanol. In an optimised experiment, a reaction mixture comprising 0.65 mmol of vanillyl alcohol, 50 mg of the activated catalyst, 2.5 mL of acetonitrile, 1.1 mmol of ^*t*^BuOOH, and 0.65 mmol of TEMPO radical was heated to 60 °C for a duration of 8 hours. The reaction temperature of 60 °C was chosen to ensure a moderate and mild environment, maintaining the stability of the MOF and preventing its decomposition. Quantitative ^1^H NMR spectra were used to determine the product of the reaction, with toluene used as the internal standard. The ^1^H NMR spectra of the reaction solutions catalysed by OP−Au@UiO‐66(Ce) are shown in Figure S12.

The redox activity of Au NP‐loaded UiO‐66(Ce) samples synthesised using double‐solvent/reduction and one‐pot methods was compared for the oxidation of vanillyl alcohol. To establish a baseline for comparison, control experiments were conducted without any catalyst and with bare UiO‐66(Ce). As shown in Table 1, a significant improvement in activity was achieved by the Au‐loaded UiO‐66(Ce) catalyst compared to the MOF alone. The MOF alone, which lacks Au nanoparticles, exhibited activity levels similar to those observed in the absence of any catalyst, with a vanillin yield of less than 5 %, indicating that the blank MOF itself does not possess significant catalytic activity for the oxidation of vanillyl alcohol. It is also important to note that with any catalyst (Entry 1 in Table 1) TEMPO by itself is not sufficient to drive the oxidation process effectively, and gives less than 2 % vanillin yield.

**Table 1:** Catalytic oxidation of vanillyl alcohol to vanillin^1^.

Entry	Catalyst	Vanillyl alcohol conversion (%)	Vanillin yield (%)	Selectivity (%)
1	Blank	4.4	1.8	40.9
2	UiO‐66(Ce)	5.0	4.5	90.4
3	OP−Au@UiO‐66(Ce)	42.8	38.5	90.1
4	3 %Au/UiO‐66(Ce)	36.8	22.7	61.8
5	0.5 %Au/UiO‐66(Ce)	25.3	13.2	52.0

^1^Reaction conditions: vanillyl alcohol (0.65 mmol), catalyst (50 mg), TEMPO (0.65 mmol), ^*t*^BuOOH (1.1 mmol), acetonitrile (2.5 mL), 60 °C, 8 h.

The one‐pot synthesised OP−Au@UiO‐66(Ce) exhibited the highest redox activity, yielding 38.5 % vanillin with 90 % selectivity. This result surpasses the vanillin yield of 22.7 % and 61.8 % selectivity achieved by the 3 %Au/UiO‐66(Ce) sample synthesised by the double‐solvent followed by reduction method. These findings suggest that encapsulating Au nanoparticles within the pores of the MOF at the point of MOF synthesis leads to improved redox activity. This may be because the two‐step process results in extrusion of Au from the MOF, as seen by PXRD which shows growth of Au particles on extended heating under reducing conditions. It is notable also that the material prepared in two steps also has PXRD pattern with no significant loss of intensity of the low angle diffraction peaks, which also suggests that most of the Au is at the surface of the material rather than within the pores. The lower conversion and selectivity observed in the 0.5 %Au/UiO‐66(Ce) sample compared to the 3 % loaded sample can be attributed to the lower concentration of active sites in the former. Table S5 provides an overview of other notable catalysts employed in this reaction. It is noteworthy that no MOF materials had been tested in this specific oxidation reaction prior to this study.

**Figure 7:**
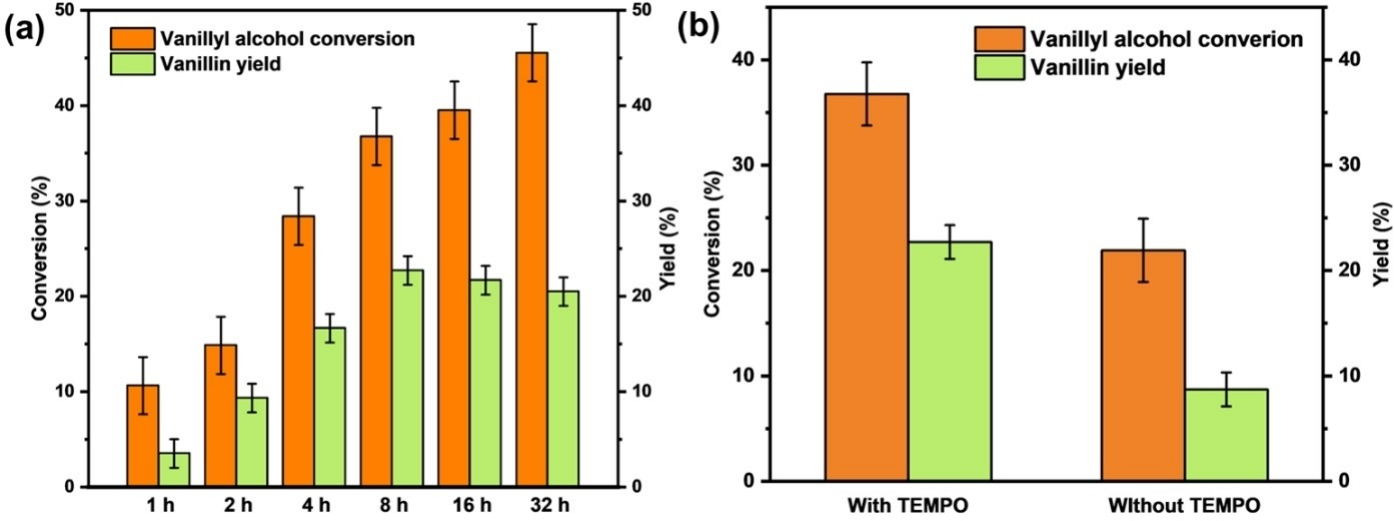
(a) Results of the oxidation of vanillyl alcohol to vanillin using 3 %Au/UiO‐66(Ce) over various reaction times. Reactions were carried out for durations ranging from 1 to 32 hours at 60 °C. ^*t*^BuOOH was used as the oxidant and TEMPO as the cocatalyst. (b) Results comparing the oxidation of vanillyl alcohol to vanillin with and without TEMPO (1 equivalent). Catalyst: 3 %Au/UiO‐66(Ce). Reaction conditions: heating at 60 °C for 8 hours with ^*t*^BuOOH as the oxidant. The error bars represent systematic errors determined by repeated analysis.

The effects of different reaction durations were investigated by conducting test reactions ranging from 1 hour to 32 hours using 3 %Au/UiO‐66(Ce) as catalyst, providing insights into the kinetics of the reaction (Figure 7(a)). The data show a significant increase in the formation of vanillin as the reaction time is extended from 1 hour to 8 hours. After 8 hours, the yield of vanillin plateaus at approximately 20 %, suggesting the establishment of an equilibrium state. While the yield plateaus, the conversion of vanillyl alcohol continues to increase. This could be due to the occurrence of side reactions or the degradation of vanillyl alcohol into unidentified products under prolonged reaction conditions, resulting in lower overall selectivity. Figure 7(b) shows a significant impact when 1 equivalent of TEMPO is added to the reaction mixture during the oxidation of vanillyl alcohol using 3 %Au/UiO‐66(Ce) as catalyst. The inclusion of TEMPO resulted in a substantial increase in both the conversion of vanillyl alcohol and the yield of vanillin, rising from approximately 10 % to 20 %. The results of the temperature‐controlled experiment (Figure S11) show that increasing the reaction temperature from 60 °C to 90 °C leads to a continued rise in the conversion of vanillyl alcohol, indicating a faster reaction rate. However, the yield of vanillin remains relatively constant, resulting in lower selectivity.

**Figure 8:**
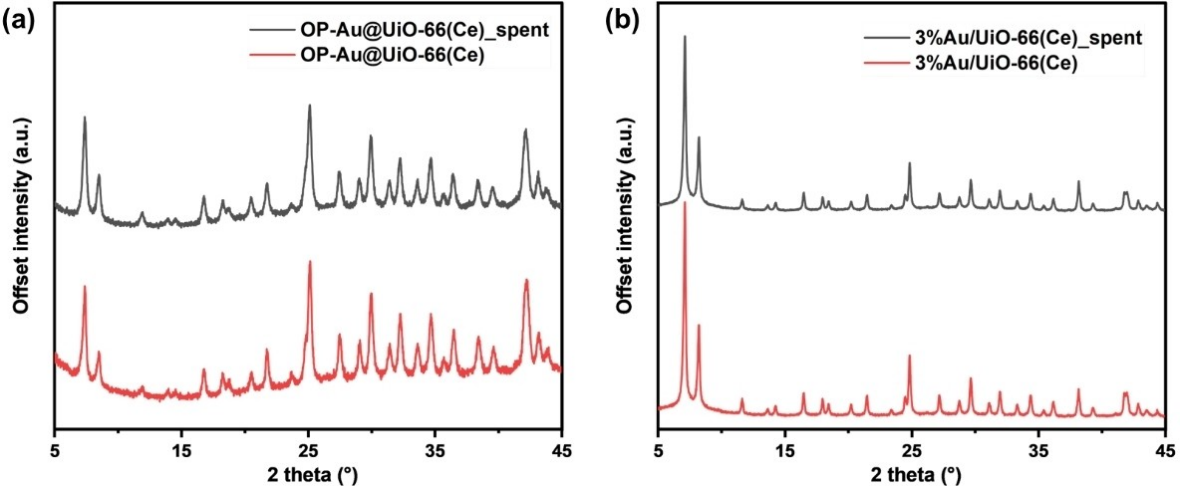
PXRD patterns of the as‐synthesised catalysts and the same MOF after the oxidation reaction for (a) OP−Au@UiO‐66(Ce) and (b) 3 %Au/UiO‐66(Ce).

PXRD patterns (Figure 8) of the as‐synthesised catalysts and the same material after the catalysis reaction were examined for both one‐pot and double‐solvent/reduction method synthesised materials. The results indicate no decrease in crystallinity and no evidence of Au nanoparticle agglomeration in the samples, suggesting that the framework structure of the materials from both synthesis methods remains intact after the catalytic reaction at 60 °C, demonstrating their robustness and stability without significant degradation. Nitrogen adsorption isotherms measured after catalysis for both samples OP−Au@UiO‐66(Ce) and 3 %Au/UiO‐66(Ce), also demonstrate the stability of the materials (Figures S14 and S15) with surface areas and porosity maintained. The minor change in BET surface area is attributed to the reagent blocking the channels. The lattice parameters of the OP−Au@UiO‐66(Ce) and 3 %Au/UiO‐66(Ce) after the catalytic reaction are 21.4847(13) Å and 21.5035(5) Å, respectively, compared to 21.4662(11) Å and 21.4926(4) Å for the fresh catalysts. The Pawley fitted patterns are shown in Figure S14–S16 and the fitting results are shown in Table S4. The recyclability of the most active catalyst, OP−Au@UiO‐66(Ce), was evaluated after each reaction cycle. After each cycle, the catalyst was reactivated by degassing at 160 °C for 2 hours. As shown in Figure S13, the catalyst demonstrated favourable recyclability with no significant decline in activity over four successive cycles. Consistent conversion of vanillyl alcohol and stable vanillin yield indicate that the catalyst maintains activity and stability, making it suitable for repeated use. Table S5 provides an overview of notable catalysts employed in this reaction. Previous studies have demonstrated that a basic environment and extended reaction time (17 hours) for Pd/SBA‐15,^[52]^ as well as molecular O₂ (20 bar) as oxidant for Fe‐doped ceria,^[53]^ are necessary to achieve high catalytic activity. In contrast, our proposed mild reaction conditions, using TEMPO as a cocatalyst and heating at 60 °C for 8 hours, achieve relatively high conversion and selectivity towards vanillin. Notably, our work is the first to test MOF‐based materials in this specific oxidation reaction, highlighting the potential for further study.

## Conclusions

A one‐pot synthesis method facilitated the simultaneous formation of Au nanoparticles and crystallisation of the UiO‐66(Ce) MOF, enabling direct encapsulation of Au nanoparticles within the MOF structure. This process resulted in a homogeneous distribution of Au nanoparticles, with a distribution of sizes centred on ~4 nm in diameter, throughout the UiO‐66(Ce) framework. The redox activity of Au/UiO‐66(Ce) materials was evaluated for the oxidation of vanillyl alcohol to vanillin, the first time a MOF catalyst has been used for this transformation. Among the tested samples, the one synthesised using the one‐pot method demonstrated the highest redox activity, having 43 % conversion with 90 % selectivity towards vanillin, compared to 37 % conversion and 62 % selectivity for the sample prepared by the double‐solvent/reduction method. Our work indicates that incorporating Au NPs within the pores of the UiO‐66(Ce) MOF, rather than loading them onto the surface, leads to improved redox activity. The one‐pot synthesised OP−Au@UiO‐66(Ce) catalyst demonstrated stability and reusability in the oxidation reaction, representing a significant advancement in sustainable vanillin production under mild conditions with potential for wide‐ranging industrial applications. The advantage of supporting gold nanoparticles is prevention of their agglomeration, as well as ease of separation of the catalysts from the reaction mixture. The catalyst we have developed could be applicable to other oxidation reactions where a heterogeneous catalyst is required using mild conditions.

## Experimental Methods

### Materials

Ammonium cerium nitrate (98.5 %, (NH_4_)_2_Ce(NO_3_)_6_, Merck), zirconium oxynitrate hydrate (99 %, ZrO(NO_3_)_2_⋅xH_2_O, Sigma‐Aldrich), benzene‐1,4‐dicarboxylic acid (98 %, H_2_BDC, Sigma‐Aldrich), formic acid (96 %, HCOOH, Merck), gold(III) chloride hydrate (99.999 % trace metals basis, HAuCl_4_⋅xH_2_O, Sigma‐Aldrich), polyvinylpyrrolidone, average M_w_~29,000 (PVP, Sigma‐Aldrich), methanol (99.8 %, VWR), 4‐hydroxy‐3‐methoxybenzyl alcohol (98 %, vanillyl alcohol, Sigma‐Aldrich), 2,2,6,6‐tetramethylpiperidinooxy, free radical (98 %, TEMPO, Thermo Scientific Chemicals) and *tert*‐butyl hydroperoxide solution (5.0–6.0 M in decane, ^*t*^BuOOH, Sigma‐Aldrich) were used as provided without further treatments.

### Synthesis

#### Scaled‐up Synthesis of UiO‐66(Ce)

The scaled‐up synthesis of UiO‐66(Ce) was adapted from a previously established method^[54]^ to allow for a 20‐fold increase in scale. The synthesis was conducted in a 250 mL Radleys Ready™ reactor, equipped with precise temperature control, via a circulating oil jacket, and reflux capabilities. Firstly, 4.1535 g (0.025 mol) of benzene‐1,4‐dicarboxylic acid (H_2_BDC) was introduced into the reactor and dissolved in 150 mL of DMF. The mixture of solvent and organic linker was pre‐heated to 100 °C in the reactor. Subsequently, a 40 mL aqueous solution containing 10.9652 g (0.020 mol) of (NH_4_)_2_Ce(NO_3_)_6_ was added to the reactor. The reaction mixture was subjected to continuous stirring under reflux conditions at 100 °C for 20 minutes. The resulting precipitate was isolated via filtration. The solid product was then washed three times with DMF and four times with acetone to remove any unreacted ligand precursor or residual impurities. The as‐synthesised UiO‐66(Ce) was immersed in 100 mL of acetone for 8 hours, followed by immersion in 100 mL of dichloromethane (DCM) for an additional 8 hours to facilitate solvent exchange. The solvent‐exchanged UiO‐66(Ce) was then subjected to overnight heating at 100 °C to evaporate the DCM.

#### Double‐Solvent Method to Immobilise Au NPs

The as‐synthesised UiO‐66(Ce) was transferred to a vacuum oven and heated under vacuum at 120 °C for 24 hours. For the loading of gold precursor, 100 mg of activated UiO‐66(Ce) was dispersed in a beaker containing 20 mL of *n*‐hexane. The mixture was subjected to sonication for 15 minutes, followed by stirring for an additional hour. Then, 0.2 mL of 30 mg/mL HAuCl_4_ hydrate aqueous solution was added dropwise under continuous stirring. The resulting powder was dried in air overnight and then degassed at 120 °C under vacuum. This initial HAuCl_4_‐loaded sample was labelled as 3 %HAuCl_4_/UiO‐66(Ce). For comparison, a lower loading sample, designated as 0.5 %HAuCl_4_/UiO‐66(Ce), was prepared by adding 0.2 mL of 5 mg/mL HAuCl_4_ aqueous solution following the same procedure. The reduction of precursor HAuCl_4_/UiO‐66(Ce) to Au nanoparticles was performed under a 5 % H_2_ in N_2_ atmosphere at 130 °C for 2 hours. Following the reduction process, the samples were designated as 3 %Au/UiO‐66(Ce) and 0.5 %Au/UiO‐66(Ce), respectively.

#### One‐Pot Synthesis of Au@UiO‐66(Ce)

In a 100 mL two‐neck round‐bottom flask, 0.4984 g (3 mmol) of H_2_BDC and 2 g of polyvinylpyrrolidone (PVP, average Mw ~29,000) were dissolved in a solvent mixture of 20 mL of dimethylformamide (DMF), 4 mL of formic acid, and 12 mL of methanol. The solution was subjected to sonication for 15 minutes to ensure thorough mixing, and then heated to 100 °C in an oil bath under reflux conditions. After 10 minutes of reflux, 20 mg of HAuCl_4_ dissolved in 1.5 ml of water was injected into the solution, causing a colour change in the solution from light yellow to brown. The reaction was allowed to continue for an additional 15 minutes. Subsequently, a solution containing 1.3703 g (2.5 mmol) of (NH_4_)_2_Ce(NO_3_)_6_ dissolved in 10 mL of water was injected into the reaction mixture and the reaction proceeded for another 20 minutes to allow the formation of the Au@UiO‐66(Ce). Upon completion, the resulting powder was collected by centrifugation. The solid was washed three times with DMF and four times with acetone to remove any unreacted ligands or impurities. Finally, the product was dried at 70 °C overnight. This sample was designated as OP−Au@UiO‐66(Ce).

### Characterisation

PXRD measurements were performed using a Panalytical Empyrean diffractometer with copper Kα_1/2_ radiation. Diffraction data were recorded between 5° and 50° 2θ. The GSAS‐II software was used to perform Pawley fits of the PXRD patterns to determine unit cell parameters.^[55]^ VT‐PXRD measurements were performed using a Bruker D8 diffractometer with Cu Kα radiation and a VÅNTEC‐1 high‐speed detector. The sample was placed inside an Anton Paar XRK 900 chamber, which was controlled by a TCU 750 temperature unit. 5 % H_2_ in N_2_ was flowed over the sample throughout the scan. The morphology and elemental mapping of the samples were measured using a JEOL 2100 TEM equipped with LaB_6_ operating at 200 kV and JEOL ARM200F TEM/scanning TEM (STEM) with a Schottky gun both at 80 kV. Annular dark‐field (ADF) STEM measurement was performed in ARM200F, with probe and image aberration CEOS correctors. ADF‐STEM images were obtained using a JEOL annular field detector with a probe current of approximately 23 pA, a convergence semi‐angle of ∼25 mrad, and an inner angle of 45–50 mrad. An Oxford Instruments X–MaxN 100TLE windowless silicon drift detector (SSD) was used to perform STEM‐EDX analysis. X‐ray fluorescence (XRF) data were measured using a Rigaku Primus IV wavelength‐dispersive X‐ray fluorescence spectrometer (WDXRF) equipped with a 4 kW X‐ray tube. The TGA analysis was performed using a Mettler Toledo TGA/DSC 1 instrument where samples were heated in air from 25 to 1000 °C with a heating rate of 10 °C/min. N_2_ adsorption isotherms were measured at 77 K using a Micromeritics ASAP 2020 apparatus, with the sample pretreated under vacuum at 120 °C for 4 h to remove any adsorbed gases or volatile impurities. The BET method was used to calculate the surface area. Ce L_III_‐edge XANES spectra were collected at BM28 beamline (XMaS) of the European Synchrotron Radiation Facility (ESRF). The measurements were conducted in fluorescence mode from powdered samples diluted with cellulose and pressed into self‐supporting pellets, using a Si (111) double crystal monochromator was used. The data were normalised using software ATHENA.^[56]^ Small‐angle X‐ray scattering (SAXS) measurements were performed using a Xenocs Xeus 2.0 instrument with a Cu Kα source and a Dectris PILATUS3 R 300 K hybrid photon counting detector. Fitting of the SAXS data was performed in the Irena analysis package.^[57]^ The scattering was modelled using spheres with a lognormal distribution of the radius. X‐ray photoelectron spectroscopy (XPS) data were collected using a Kratos Axis Ultra DLD spectrometer with a base pressure below 1×10^−1^⁰ mbar. Measurements were conducted at room temperature with a monochromated Al Kα X‐ray source (*hν*=1486.7 eV). Data analysis was performed using CasaXPS software. ^1^H NMR measurement was performed with a Bruker Avance III HD 400 MHz instrument.

### Catalytic Oxidation Reactions

Initially, the catalyst was activated by heating it to 160 °C under vacuum for 2 hours and after cooling the catalyst to room temperature, it was immediately used in the reaction. In a 7 mL glass Pyrex reaction tube, 100 mg (0.65 mmol) of vanillyl alcohol and 50 mg of the activated catalyst were added to 2.5 mL of acetonitrile. Subsequently, 0.2 mL of ^*t*^BuOOH solution (5.0–6.0 M in decane) and 100 mg (0.65 mmol) of TEMPO as cocatalyst were introduced into the mixture. The reaction mixture was sealed and subjected to sonication for 10 minutes. Then, the reaction tube was transferred to a pre‐heated oil bath maintained at 60 °C and heated for 8 hours under vigorous stirring. Once the reaction mixture cooled to room temperature, toluene as an internal standard, was accurately weighed and thoroughly mixed into the solution. The mixture was then subjected to centrifugation to separate the solution from the solid catalyst. The resulting solution was filtered and transferred to an NMR tube and subsequently diluted with CDCl_3_. Before each recyclability test, the used catalyst was washed four times with acetone, dried overnight at 70 °C, and reactivated by degassing at 160 °C for 2 hours.

^1^H NMR spectroscopy was used to quantify the product of the reaction. Samples were spiked with a known exact molar quantity of an internal standard (toluene). The molar quantity of vanillin $m_{Vanillin}$ in the sample was calculated using Equation (1) as shown below:

(1) $$m_{Vanillin}=\;\frac{I_{analyte}\;\times \;P_{standard}}{I_{standard}\;\times \;P_{analyte}}\;\times m_{standard}$$

where $m_{standard}$ is the known molar amount of the internal standard, $I_{analyte}$ and $I_{standard}$ represent the integral of the resonance peaks for the analyte and the standard, and $P_{standard}$ and $P_{analyte}$ are the numbers of protons corresponding to the resonance peak.

The conversion rates of vanillyl alcohol (VA) and the yields of vanillin were calculated using the following equations:

(2) $$Conversion=\;\frac{VA\;initial\;moles-VA\;final\;moles}{VA\;initial\;moles}\;\times 100\;\%$$

(3) $$Yield\;=\frac{moles\;of\;vanillin\;formed}{VA\;initial\;moles}\;\times 100\;\%$$

## Conflict of Interests

The authors declare no conflict of interest.

1

## Supporting information

As a service to our authors and readers, this journal provides supporting information supplied by the authors. Such materials are peer reviewed and may be re‐organized for online delivery, but are not copy‐edited or typeset. Technical support issues arising from supporting information (other than missing files) should be addressed to the authors.

Supporting Information

## Data Availability

The data that support the findings of this study are available from the corresponding author upon reasonable request.
