# Supplementary material for: Cerium‐Organic Framework UiO‐66(Ce) as a Support for Nanoparticulate Gold for Use in Oxidation Catalysis
Source: Chem Asian J. 2024 Oct 31;19(24):e202401035. doi: 10.1002/asia.202401035 (PMC11639647; doi:10.1002/asia.202401035)
Supplement: Supplementary file 1 — Supporting Information [file ASIA-19-e202401035-s001.pdf]

Chemistry - An Asian Journal

Supporting Information

Cerium-Organic Framework UiO-66(Ce) as a Support for Nanoparticulate Gold for Use in Oxidation Catalysis

Baiwen Zhao, Reza J. Kashtiban, Steven Huband, Marc Walker, and Richard I. Walton*

Supporting Information

Cerium-Organic Framework UiO-66(Ce) as a Support for Nanoparticulate Gold for Use in Oxidation Catalysis

Baiwen Zhao,[1] Reza J. Kashtiban,[2] Steve Huband,[2] Marc Walker,[2] and Richard I. Walton[1]*

1. Department of Chemistry, University of Warwick, Gibbet Hill Road, Coventry CV4 7AL, UK
2. Department of Physics, University of Warwick, Gibbet Hill Road, Coventry CV4 7AL, UK

*Author for correspondence: r.i.walton@warwick.ac.uk

**Section**	**Content**	**Page**
S1	Chemical and thermal studies	S2
S2	N_2 sorption measurements	S4
S3	XPS studies	S6
S4	Catalysis studies	S8

S1: Chemical and thermal studies

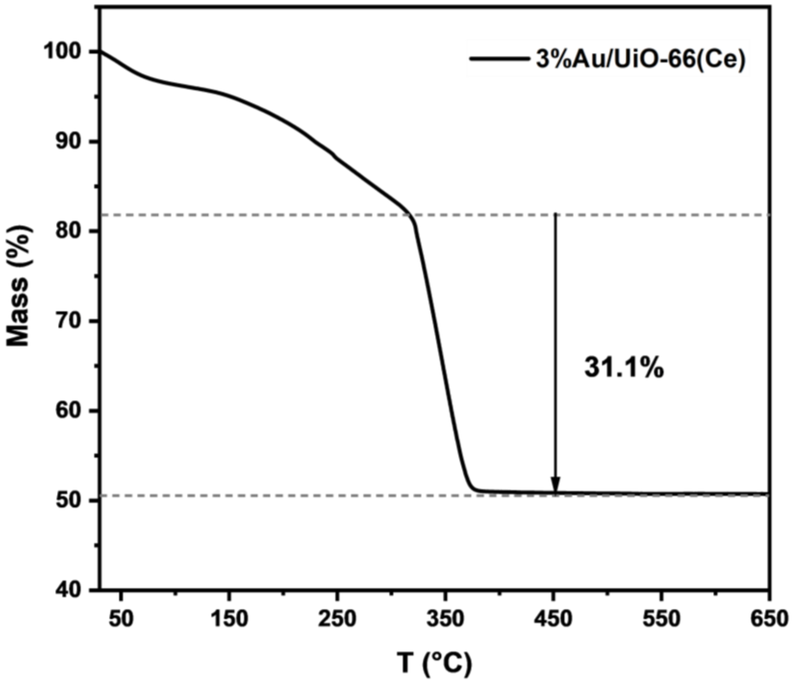

Figure S1. TG curve of the synthesised OP-Au@UiO-66(Ce) in air

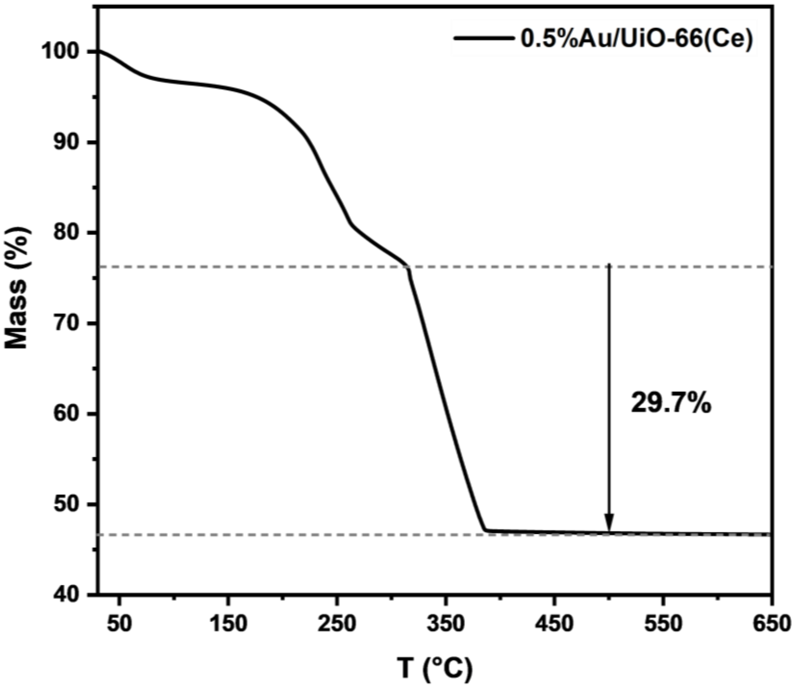

Figure S2. TG curve of the synthesised 0.5%Au/UiO-66(Ce) in air

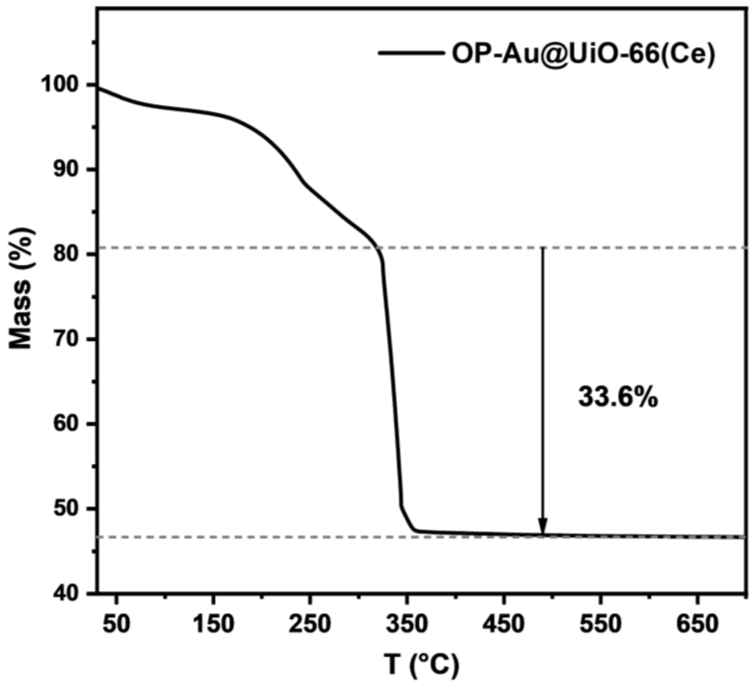

Figure S3. TG curve of the synthesised OP-Au@UiO-66(Ce) in air

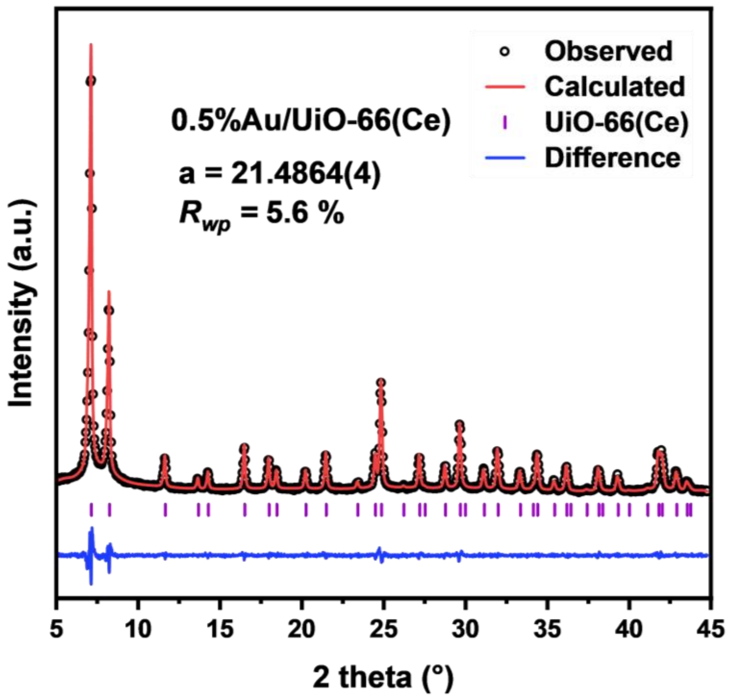

Figure S4. PXRD pattern of synthesised 0.5%Au/UiO-66(Ce) refined using a Pawley fit (Space group $Fm\bar{3}m$). No Au was observed, so the pattern was fitted in the single UiO-66 phase.

Table S1. Weight percentages of the Au loading on the MOF measured by XRF

Sample	Au wt. /% by XRF
0.5%Au/UiO-66(Ce)	0.57
3%Au/UiO-66(Ce)	3.20
OP-Au@UiO-66(Ce)	3.86

S2: N_2 sorption measurements

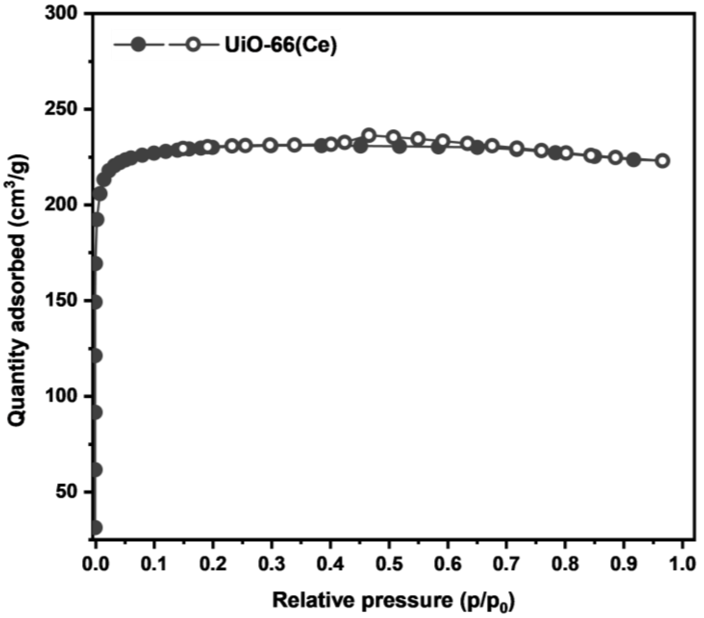

Figure S5. N_2 adsorption-desorption isotherm plots at 77K of UiO-66(Ce) support. Filled symbols mark the adsorption, while empty symbols mark the desorption step.

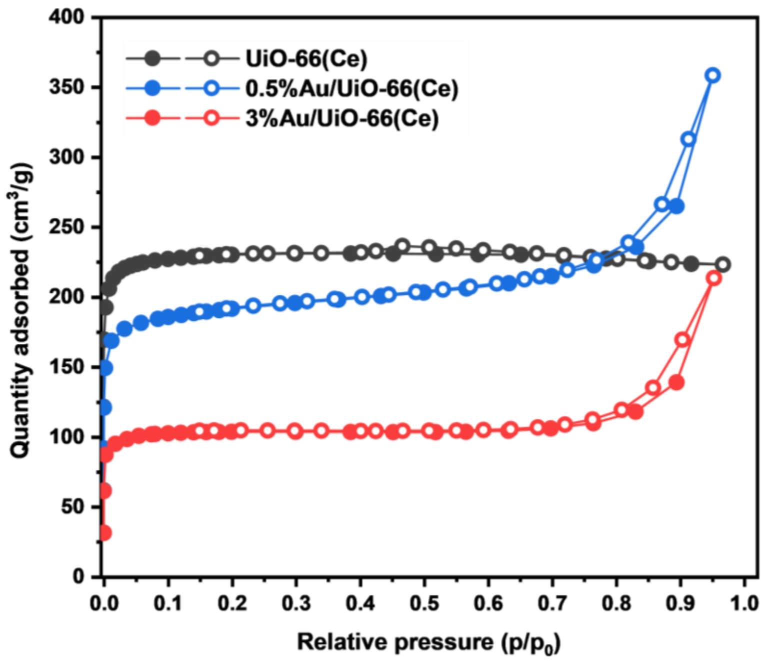

Figure S6. N_2 adsorption-desorption isotherm plots at 77K of OP-Au@UiO-66(Ce) compared to the UiO-66(Ce) support. Filled symbols mark the adsorption, while empty symbols mark the desorption step.

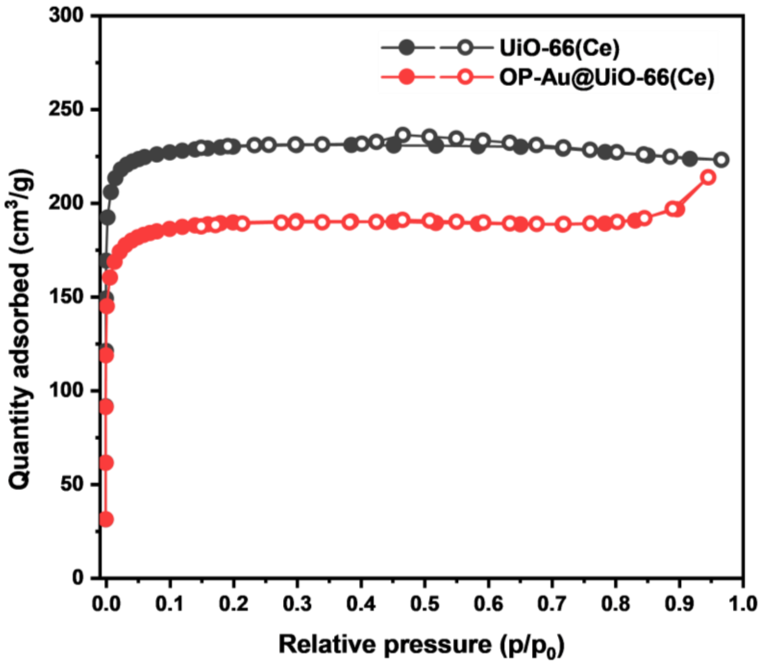

Figure S7. N_2 adsorption-desorption isotherm plots at 77K of 3%Au/UiO-66(Ce), 0.5%Au/UiO-66(Ce) compared with the UiO-66(Ce) support. (curve in hollow symbol represents the desorption branch)

Table S2. BET surface area and micropore volume of the Au loaded UiO-66(Ce) and the blank MOF obtained from the isotherms (Figure S5-S7)

**Sample**	**BET surface area (m^2/g)**	**Micropore volume (cm^3/g)**
UiO-66(Ce)	827	0.33
OP-Au@UiO-66(Ce)	680	0.26
0.5%Au/UiO-66(Ce)	683	0.24
3%Au/UiO-66(Ce)	368	0.15

S3: XPS studies

Table S3. XPS Analysis of 3%Au/UiO-66(Ce), 3%$HAuCl_4$/UiO-66(Ce) and OP-Au@UiO-66(Ce).

		**Sample**		
		3%Au/UiO-66(Ce)	3%HAuCl4/UiO-66(Ce)	OP-Au@UiO-66(Ce)
**Peak Position (eV)**	Au(0)	84.9	84.2	84.7
	Au(I)	86.1	85.4	85.9
	Au(III)	87.4	86.7	87.0
**Proportion (at. %)**	Au(0)/Au	29	12	26
	Au(I)/Au	63	47	51
	Au(III)/Au	8	41	23
	Ce(III)/Ce	41	36	33
	Ce(IV)/Ce	59	64	67

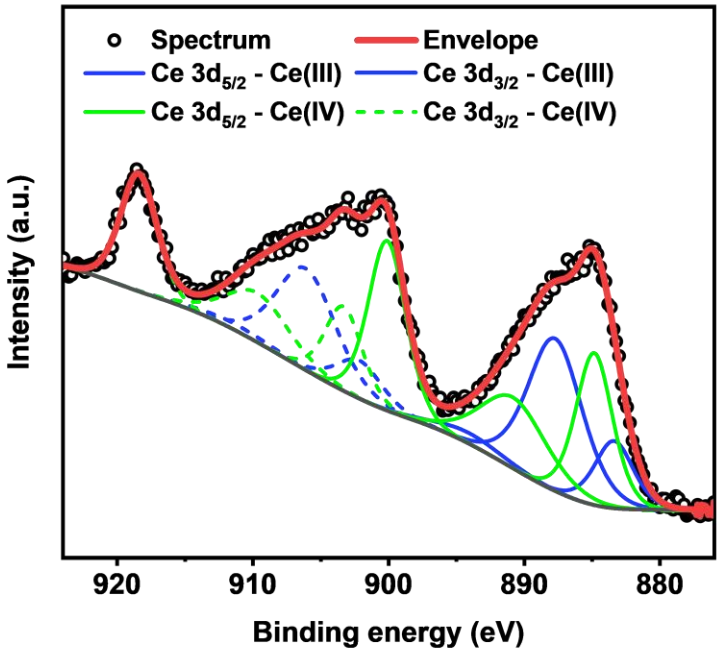

Figure S8. XPS spectra of Ce 3d for 3%$HAuCl_4$/UiO-66(Ce)

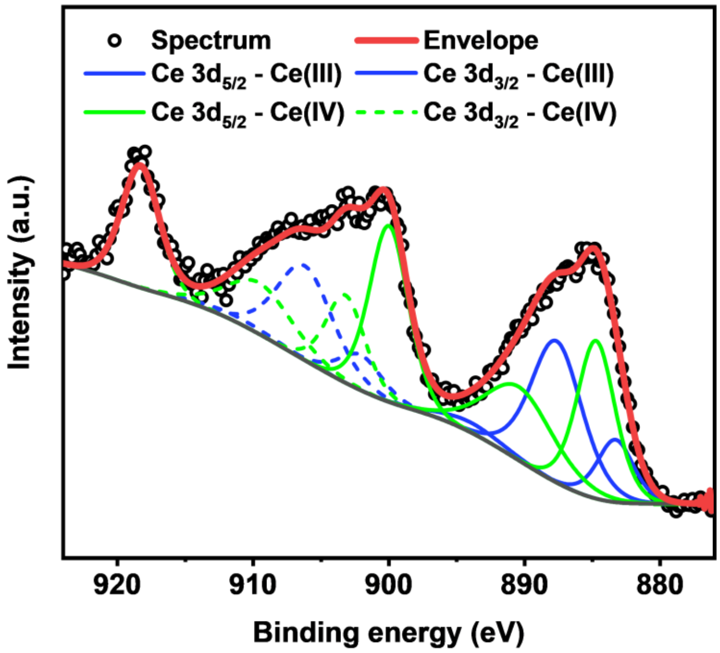

Figure S9. XPS spectra of Ce 3d for 3%Au/UiO-66(Ce)

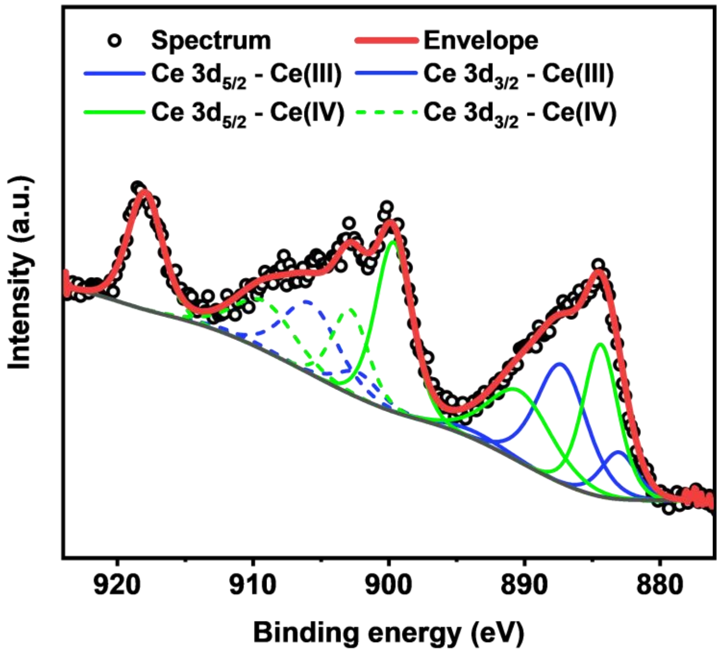

Figure S10. XPS spectra of Ce 3d for OP-Au@UiO-66(Ce)

S4: Catalysis studies

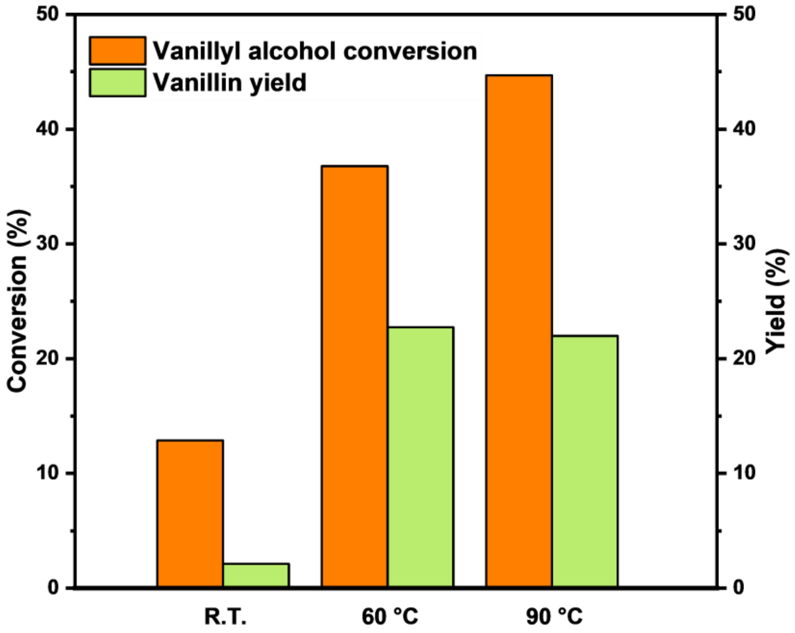

Figure S11. Comparison of the activity in oxidation of vanillyl alcohol to vanillin at room temperature, 60 °C and 90 °C. Catalyst: 3%Au/UiO-66(Ce). Reactions condition: Heating for 8 hours, ^{t}BuOOH was used as oxidant, and TEMPO (1 equivalent) as co-catalyst.

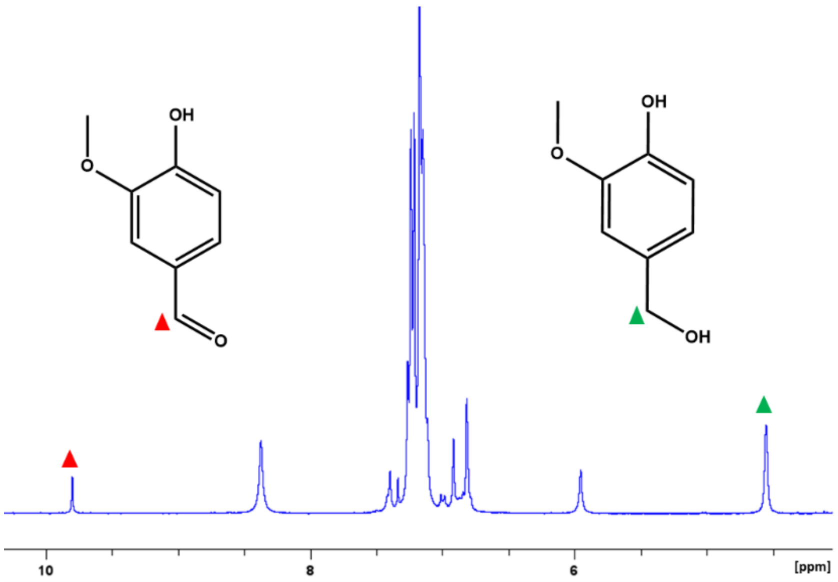

Figure S12. ^{1}H NMR spectra of post experimental solution of the reaction catalysed by OP-Au@UiO-66(Ce). Catalyst: OP-Au@UiO-66, TEMPO (1 equivalent) as co-catalyst. Reactions condition: *t*BuOOH as oxidant, 60°C, 8 hours. Resonance peaks are assigned for vanillyl alcohol and vanillin.

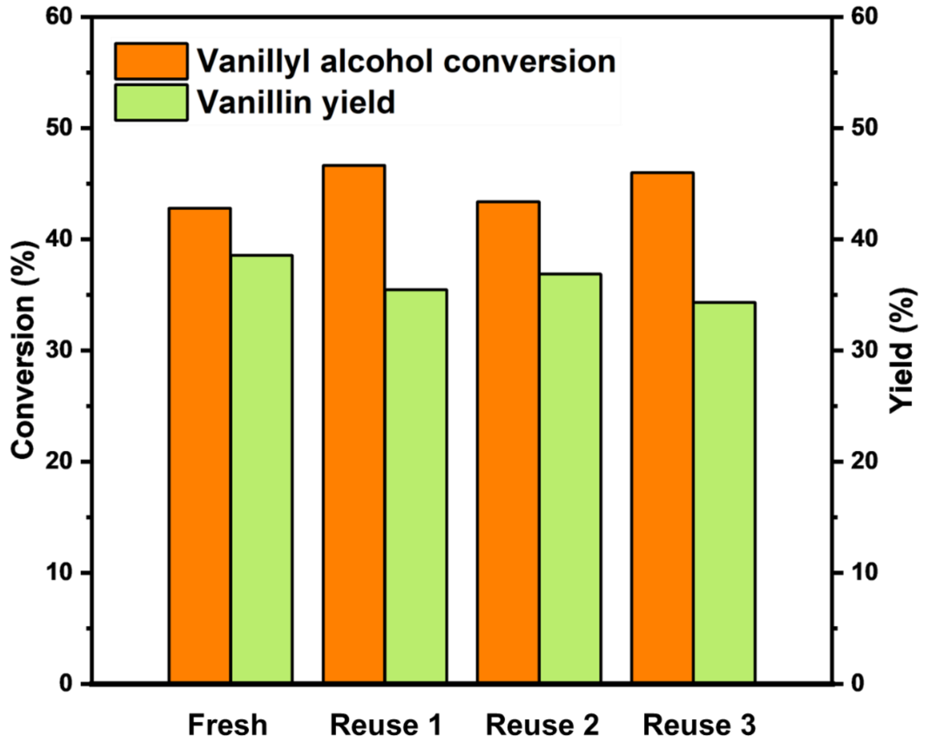

Figure S13. Results of catalytic oxidation of vanillyl alcohol by OP-Au@UiO-66(Ce) across four consecutive reaction cycles reusing the same catalyst.

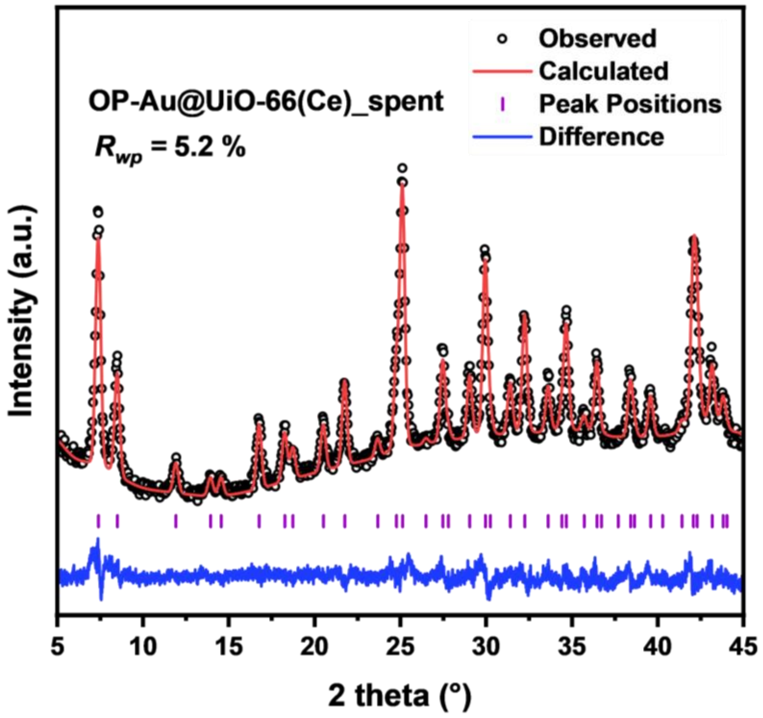

Figure S14. Pawley plot of OP-Au@UiO-66(Ce) catalyst after catalytic reaction

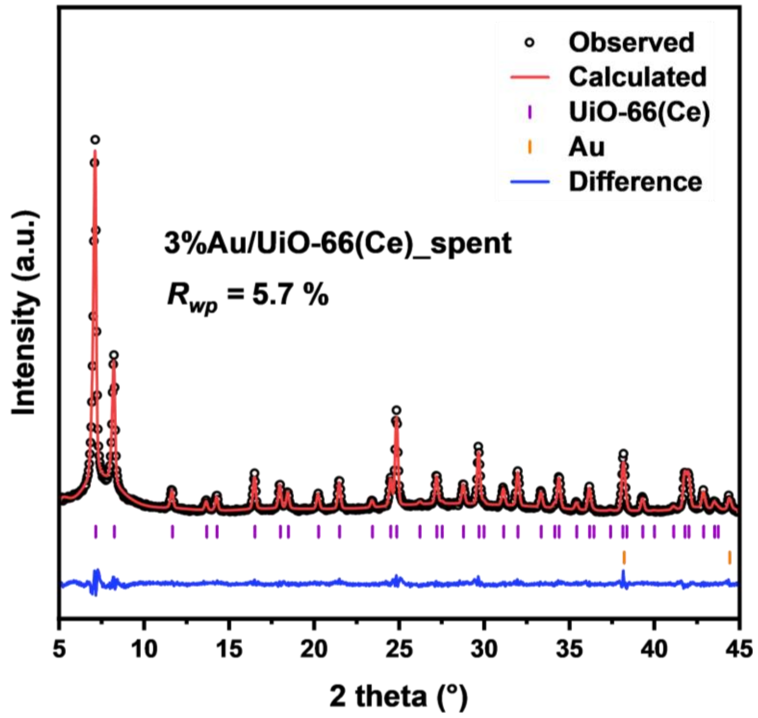

Figure S15. Pawley plot of 3%Au/UiO-66(Ce) catalyst after catalytic reaction.

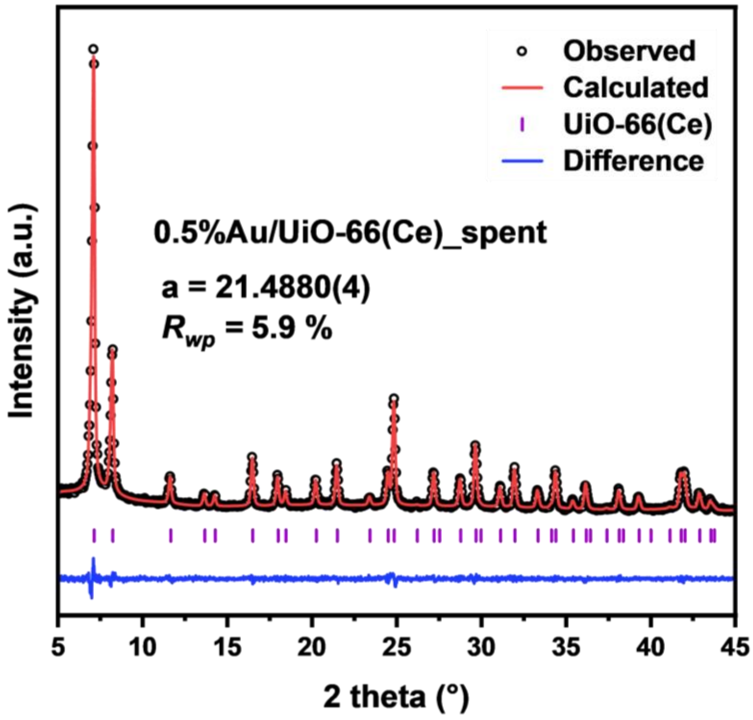

Figure S16. Pawley plot of 0.5%Au/UiO-66(Ce) catalyst after catalytic reaction

Table S4. Lattice parameters of the UiO-66(Ce) support as well as the materials before and spent from catalytic reactions. Results are obtained from Pawley fits. (Figure 1(a), 3(a), 5(a) and Figure S11-S13)

Compound	*a* (Å)	α (°)	Space group	R_{wp} (%)	GoF
UiO-66(Ce)	21.4712(4)	90	$Fm\bar{3}m$	3.5	1.16
OP-Au@UiO-66(Ce)	21.4662(11)	90	$Fm\bar{3}m$	5.3	1.44
0.5%Au/UiO-66(Ce)	21.4864(4)	90	$Fm\bar{3}m$	5.6	1.41
3%Au/UiO-66(Ce)	21.4926(4)	90	$Fm\bar{3}m$	4.9	1.18
OP-Au@UiO-66(Ce)_spent	21.4847(13)	90	$Fm\bar{3}m$	5.2	1.78
0.5%Au/UiO-66(Ce)_spent	21.4880(4)	90	$Fm\bar{3}m$	5.9	1.17
3%Au/UiO-66(Ce)_spent	21.5035(5)	90	$Fm\bar{3}m$	5.7	1.32

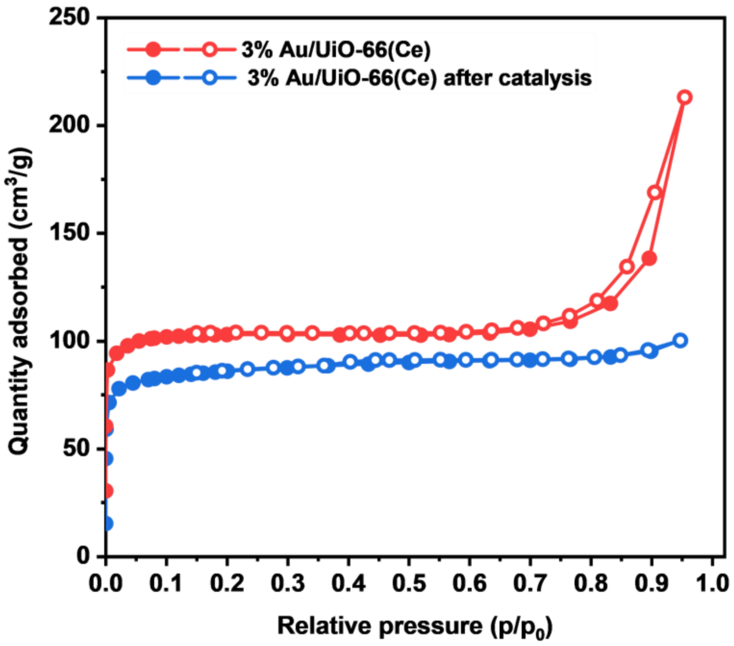

Figure S17. N_2 adsorption-desorption isotherm plots at 77K of 3%Au/UiO-66(Ce) before (BET surface area = 368 m^2/g) and after (BET surface area = 293 m^2/g) catalysis. (curve in hollow symbol represents the desorption branch)

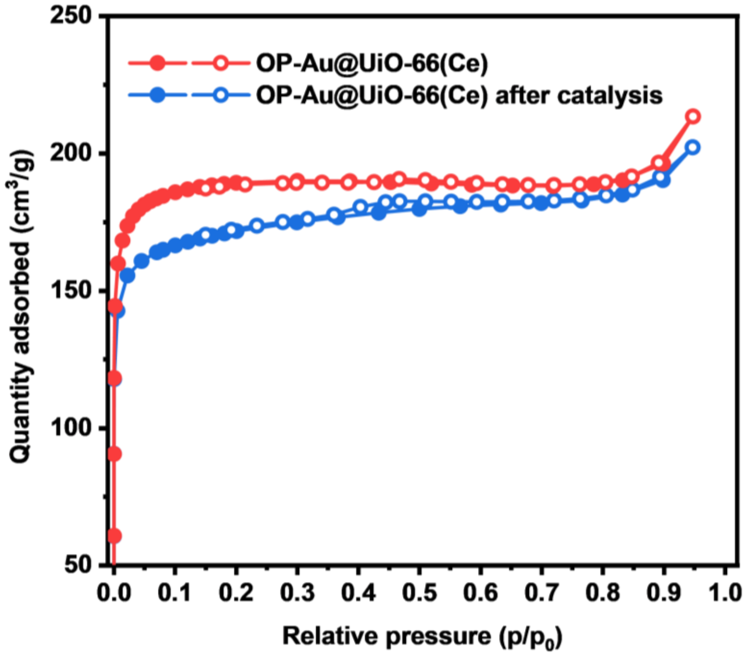

Figure S18. N_2 adsorption-desorption isotherm plots at 77K of 3%Au/UiO-66(Ce) before (BET surface area = 680 m^2/g) and after (BET surface area = 593 m^2/g) catalysis. (curve in hollow symbol represents the desorption branch)

Table S5. Catalytic activity of oxidation of vanillyl alcohol to vanillin over different catalysts. VA = vanillyl alcohol, Sel = selectivity

Entry	Catalyst	Solvent	Oxidant	Temp (°C)	Time	VA conv (%)	Sel (%)	Ref
1	OP-Au@UiO-66(Ce)	Acetonitrile	^{t}BuOOH	60	8 h	43	90	This work
2	Pd/ SBA-15 /K_2CO_3	Water	No oxidant	80	17 h	100	99	52
3	$Ce_{0.8}Fe_{0.2}O_2$	Acetonitrile	20 bar O_2	140	5 h	91	99	53
4	CeO_2	Acetonitrile	20 bar O_2	140	5 h	40	95	53
5	Fe_2O_3	Acetonitrile	20 bar O_2	140	5 h	25	97	53
6	Co_3O_4 /NaOH	Isopropanol	6.8 bar air	80	6 h	80	98	58
7	Co_3O_4	Water	H_2O_2	75	1 h	32	22	59
8	$Ce_{0.8}Zr_{0.2}O_2$	Acetonitrile	20 bar O_2	140	5 h	98	99	60
9	$CoMn_2O_4$	Acetonitrile	21 bar air	140	2 h	62	83	61
10	$Cu_{1.5}Mn_{1.5}O_4$ / NaOH	Isopropanol	H_2O_2	95	2.5 h	94	99	62

References

52. W. Fu, L. M. Yue, X. G. Duan, J. Li and G. Z. Lu, *Green Chem.*, 2016, **18**, 6136-6142.
53. S. Palli, Y. Kamma, S. Nazeer, B. M. Reddy and T. V. Rao, *Res. Chem. Intermed.*, 2022, **48**, 4579-4599.
58. A. Jha and C. V. Rode, *New J. Chem.*, 2013, **37**, 2669-2674.
59. R. Behling, G. Chatel and S. Valange, *Ultrason. Sonochem.*, 2017, **36**, 27-35.
60. P. Reddy, B. G. Rao, T. V. Rao and B. M. Reddy, *Catal. Lett.*, 2019, **149**, 533-543.
61. A. Jha, K. R. Patil and C. V. Rode, *ChemPlusChem*, 2013, **78**, 1384-1392.
62. S. Saha and S. B. Abd Hamid, *RSC Adv.*, 2016, **6**, 96314-96326.
